# Challenges to Ebola preparedness during an ongoing outbreak: An analysis of borderland livelihoods and trust in Uganda

**DOI:** 10.1371/journal.pone.0230683

**Published:** 2020-03-26

**Authors:** Megan M. Schmidt-Sane, Jannie O. Nielsen, Mandi Chikombero, Douglas Lubowa, Miriam Lwanga, Jonathan Gamusi, Richard Kabanda, David Kaawa-Mafigiri

**Affiliations:** 1 Department of Social Work and Social Administration, Makerere University School of Social Sciences, Kampala, Uganda; 2 Center for Social Science Research on AIDS, Makerere University School of Social Sciences, Kampala, Uganda; 3 Department of Anthropology, Case Western Reserve University, Cleveland, Ohio, United States of America; 4 UNICEF Uganda, Kampala, Uganda; 5 Uganda Ministry of Health, Kampala, Uganda; Tulane University, UNITED STATES

## Abstract

Ebola Virus Disease in the Democratic Republic of Congo (DRC) was declared a public health emergency of international concern on July 17, 2019. The first case to cross the border into Uganda in June 2019 demonstrates the importance of better understanding border dynamics in a context of Ebola. This paper adopts a political economy approach to contextualize epidemic response programs conducted in moderate- and high-risk border districts in Uganda, through a qualitative study with 287 participants. To that end, our aim was to describe the historical underpinnings of the borderlands context; the role of livelihood strategies in constraining risk avoidance decisions; and the dynamics of trust in authority figures, including health workers. This paper reports that border communities are highly connected, for a variety of social and economic reasons. These daily realities are in direct opposition to guidance to limit travel during an active Ebola epidemic. We argue that an ability to limit movement is constrained by the economic need to seek livelihood strategies wherever that may be. Moreover, border regions are populated by communities with long-standing distrust in authority figures, particularly in fishing areas. This distrust spills over with consequences for Ebola prevention and control activities. This research indexes the importance of tailoring Ebola programming and policies to consider the political and economic dynamics of borderlands.

## Introduction

Ebola Virus Disease in the Democratic Republic of Congo (DRC) was declared a public health emergency of international concern on July 17, 2019. Prior to that, the first confirmed cases of Ebola Virus Disease (EVD) to spread beyond DRC emerged in Kasese District in Uganda on June 12, 2019. A 5-year old boy and his mother had crossed into Uganda, avoiding screening and isolation protocols at the border. This case was not unexpected in a region where communities are socially, economically, and politically integrated along a porous border. This case also underscores the importance of anthropological research in borderlands, where anthropologists can interrogate the reasons for community and individual reluctance to engage with Ebola response efforts.

Recent research has documented the broader political-economic context of (mis)trust in authorities, such as colonial history, with implications for Ebola preparedness and control [1–3]. Further, the role of mistrust has been identified as a driver of low community engagement with the Ebola response [4–5]. Research has also demonstrated the need for additional information on the context of outbreaks and epidemics such as Ebola [6]. However, research has not explicitly linked studies of border regions, or borderlands, economic livelihood strategies, and (mis)trust in authorities. This paper reports on data from a qualitative study in Uganda’s borderlands during an ongoing epidemic in the DRC. We utilize a political economy framework to describe the historical roots of livelihood strategies and (mis)trust in authorities, with implications for Ebola response efforts. Our research has implications for Ebola response efforts, which should include tailored solutions that consider political and economic context [6].

### Ebola in central Africa in 2019

While the geographic scope and scale of the EVD epidemic in DRC has now reduced greatly [7], at its peak many people were reported to be no longer seeking care and responders raised concerns about community trust in the response [8]. Cases were reported in Goma [9], which is a major transportation hub in Central Africa with linkages and routes to nearby nations. This was particularly of concern due to the porous border between the DRC, Rwanda, and Uganda, and political instability in the affected area leading to a continuous influx of refugees to Uganda, low community uptake of Ebola prevention activities in Uganda's high-risk districts, and fluid social and cultural norms related to Ebola prevention and control [10]. Meanwhile, during the epidemic, the Ugandan National Task Force for EVD Preparedness and Response cited an urgent need for a strategy for border health, cross-border cooperation, and scaled-up risk communication among other priorities [10,11]. These and future efforts must be underpinned by local community engagement [6], with an understanding of borderland dynamics. Effective local community engagement requires an understanding of the political and economic context of EVD outbreak, which anthropology is well-placed to examine.

### Political-economic context of Uganda’s borderlands

This research is situated within the anthropological literature on political economy [12–14], which places contemporary public health issues in a postcolonial, political, and economic context. We describe persistence of Ebola risk behavior not as a failure of the individual per se, but as a manifestation of key aspects of political economy in Uganda: (1) the role of British colonial policies in marginalizing certain ethnicities, such as those in this study, and (2) the postcolonial drivers of poor socioeconomic status characterized by low-wage and precarious employment. Firstly, we should acknowledge that the present-day national borders are colonial fabrications that were political and arbitrary [15]. These borders do not reflect the shared kinship and traditions by Congolese and Ugandans on both sides of the border. Additionally, Uganda’s border regions are populated by ethnic groups that were historically marginalized in British colonial Uganda. British colonial rulers privileged the Ganda Kingdom, ruling through Ganda leaders who were given positions of power over other ethnic groups [16]. These dynamics are emblematic in the case of the “Lost Counties” when British captured territory in the Nyoro Kingdom in Western Uganda was given to Ganda rulers [17,18]. This colonial privileging of one ethnic group over others has implications for contemporary tensions across Uganda [19]. Importantly, it bred tendencies to mistrust authority, which continued throughout the postcolonial era to affect uptake of public services including health services. Additionally, this framing draws on other research on the historical and political context of trust in authorities in Uganda [20,21].

Postcolonial policies in Uganda shape contemporary economic opportunity, which is limited after decades of policies that privilege economic growth over job creation [22]. Beginning in the 1980s, Uganda adopted reforms to liberalize its economy [22]. Free market policies were instituted to promote economic growth, stabilize inflation, and maintain a sustainable balance of payments [22,23]. It also saw reduced state investment in the health care system. While the country saw improved economic growth, this growth has been concentrated in the upper echelons of society as the Gini coefficient raised from 0.39 in 1996 to 0.43 in 2016 [23,24]. This economic liberalization has not translated into adequate formal job creation in the country, evidenced in the high rate of participation in the informal economy [22]. Economic opportunities in the informal economy are limited, unstable, and unregulated, contributing to broader precarity for most Ugandans [22,25]. It should be noted that the public service delivery model is the predominant form of access to health care in Uganda. Yet any significant development of health service delivery systems was principally urban-oriented and in the central regions of Uganda. As such, communities in borderlands tended to lag behind in public service delivery. Health care access is much more limited in borderlands compared to the central regions of the country [26]. Together, such historically rooted drivers of limited access to health services bred a sense of mistrust in public authority, especially in historically neglected border districts in the West.

Finally, we draw on a concept of structural violence to describe how inequality can manifest as violence and cause harm for marginalized populations such as those in the border regions. A concept of structural violence derives from a political economy approach, and examines the impact of “unequal life chances” on individual well-being [27,28]. Our research engages with Farmer and colleagues’ call [29] to document the complex workings of political and economic processes and bear witness to the harms that ensue by focusing on cost-effectiveness over well-being. In this paper, we use structural violence to describe the broader context of what it means to live in the borderlands, with implication for livelihood and well-being.

### Anthropological research on Ebola

Anthropologists have been increasingly engaged in epidemic response, for example through the Ebola Response Anthropology Platform (ERAP) [30] or the Social Science in Humanitarian Action Platform (SSHAP) [31]. Anthropologists have contributed in key areas, such as bolstering more authentic community engagement in the response [32], reframing harmful terms like “resistance” or “compliance” with response activities [33], and demonstrating the cultural importance of dignified burials [34]. Previous literature in an epidemic context demonstrates how Ebola containment efforts may hinder livelihood opportunities [35], and how livelihood strategies and coping mechanisms make individuals resilient to changes in livelihood [36]. In prior research, mistrust of health workers is contextualized to describe the low community uptake of response efforts [6,37–39]. There are also significant examples of anthropological work on the broader context of Ebola in West and Central Africa [6,40]. We build on this existing work to contextualize Ebola in Uganda’s borderlands.

## Methods

### Overview

We use an anthropological approach to describe the political and economic context of livelihood strategies and (mis)trust in the epidemic response in order to inform Ebola prevention, transmission, and preparedness. Our research engages with previous anthropological work on low community uptake of Ebola response activities and contributes to the literature on Ebola by investigating the ways in which borderland dynamics and political economy shape trust. Anthropological research was conducted in February-March 2019 in 17 border districts designated by the MOH as being at moderate-risk (Priority 2 and 3) and high-risk (Priority 1) of EVD in Uganda. The research informed the Ministry of Health National Task Force on Ebola risk communication and social mobilization (RCSM) work related to Ebola preparedness in Uganda.

### Study context

Districts were located along the border of Uganda and DRC. Districts were grouped into five cultural groupings (Table 1), namely: Bafumbira, Banyoro, Bakonzo/Batoro, Baganda and Lugbara/Alur; so that different cultural areas were sampled from to account for differences in the social and cultural context of EVD in Uganda and to capture a heterogeneity of settings.

**Table 1:** Cultural grouping of study districts.

Cultural Group	District	Region
Bafumbira	Isingiro	South-Western
	Kisoro	
	Kanungu	
	Rukungiri	
Banyoro	Kagadi	Western
	Buliisa	
	Hoima	
Batoro and Bakonzo	Kabarole	Western
	Bunyangabu	
	Ntoroko	
	Bundibugyo	
	Kasese	
	Kyenjojo	
Baganda	Luweero	Central
	Kampala	
Lugbara and Alur	Pakwach	North-Western
	Arua	

The study was primarily conducted in district offices, health facilities (public hospitals, private clinics and pharmacies), daily-livelihood spaces (market areas, local shrines, water collection points, village meeting points) and at formal and informal border crossings. In each district, research assistants met with the Chief Administrative Officer (CAO; the district administrator) and District Health Officer (DHO) to get the relevant approvals for research. All introduction and study approval letters were stamped by each district’s officials. From there, the team commenced work with an interview with the DHO or an official in the DHO's office. These interviews served as a guide to help us to hone our study sites in each district.

### Study population and sampling

The study population (*N* = 287) included youth and adults, sampled from various sectors of the community (Table 2).

**Table 2:** Study population by participant characteristics.

Study Population	FGD Participants	KII Participants
Local Leadership	9	15
District Representatives	0	13
Private and Government Health Center Staff	0	12
Civil Society Organization Staff	6	9
Religious Leaders	18	3
Religious Organization Staff	8	8
Traditional Healers	0	3
Community Youth	42	5
Community Men	54	4
Community Women	63	4
Community Influencers/Gate Keepers	0	4
Frontline Health Workers and Ebola Responders	0	5
Members of the National Task Force	0	2
**Total**	**200**	**87**

A total of 25 focus group discussions (*n* = 200 participants), key informant interviews (*n* = 87), and field notes were gathered from the 17 moderate- and high-risk districts in Uganda. Participants were sampled using a non-probability strategy, purposively selected based on cultural grouping and time spent in the community, with variable ages, gender, types of work, education levels and religion. Data was collected by the relevant research team in the local language or in English, depending on the *lingua franca* and participant’s choice.

We collected demographic information such as age, gender, type of work (e.g. what work do you do and for how long?), education level, and religion (e.g. what are some of the religious beliefs in this community?). The FGD guide was semi-structured and tailored to the type of FG participant, but covered topics such as religious and safe burial practices, caretaking practices, trust in health care workers, livelihood practices, leadership practices and rapport, collective efficacy to prevent EVD, social, cultural, and gender norms. The interviews were also semi-structured and covered a range of topics including: religious beliefs & safe burial practices, leadership practices and rapport, intra-community conflict, social norms regarding safe burials, collective efficacy to prevent EVD, social, cultural, and gender norms related to caretaking, traditional healers' understanding of EVD transmission, community trust and rapport, and incorporation of culturally-sensitive practices.

### Key measures

Two key measures, livelihoods and trust, were included in this study.

*Livelihoods* were assessed using the following domains: current employment status of participants, number of jobs and hours worked per week, consistency of employment and income, when participants crossed the border for livelihood strategies, and livelihood barriers or facilitators to health-seeking behavior and EVD preparedness.

*Trust* was assessed based on the following dimensions: community perceptions of authority figures and health workers, challenges encountered in interactions with authority figures, accessibility of individuals, and uptake of information. Our team also asked about where community members first seek health services, and whether they can access a preferred clinic or health center.

### Analysis

Qualitative data analysis employed an inductive and emergent approach, typical in thematic analysis [41]. Each code served as an indexing or measurement device to assign values to the text and help organize the data [41]. After the codebook was developed, we pilot tested it on five KII transcripts and two FGD transcripts from various cultural groupings. The coding process was iterative and ongoing and used a priori codes chosen beforehand and in vivo codes that came up throughout the analysis [41,42]. Once the codebook was finalized, multiple coders coded text from the FGDs, the KIIs, and relevant field notes. One single researcher synthesized codes into broader themes. Patterns were identified by examining the frequency of codes, code context, and how the codes could be grouped into categories. These specific themes were refined further to develop the final list of themes and theme definitions.

### Ethics statement

The study complied with international (Helsinki) and Ugandan research regulations. Approval was obtained from the Uganda National Council for Science and Technology (SS 4910) and Makerere University School of Social Sciences Research Ethics Committee (MAKSS REC 01.19.252) to ensure adherence to research protocols, procedures, and ethics, and human subjects protection. The study was also registered with the Office of the President, Research, in Uganda. Written informed consent was sought from all participants. Informed consent was documented using ethics board approved informed consent forms, which included a space for a witness signature. For minors (<18 years of age), assent forms were used which included a line for parent or guardian signature, in addition to the witness signature.

## Results

This article focuses on three major findings from this research: 1) the context of the borderland and a porous border, which facilitates constant cross-border travel; 2) the role of livelihoods and cross-border movement for economic activity; and 3) the spread of rumors and information across borders, and the exacerbation of (mis)trust in authorities that shaped mistrust in Ebola preparedness efforts.

### Border dynamics along a porous border

Participants (*n* = 61) discussed the high level of regional connectivity along the DRC-Uganda border, where large numbers of people cross the border each day. We noted mainly circular movement—Congolese come to Uganda to purchase merchandise, then return to the DRC shortly thereafter, and Ugandans go into the DRC for water or to trade at the market, and then come back shortly thereafter. Large numbers of Congolese cross into Uganda each day, seeking asylum from war or economic pressures at home. Our team observed Congolese refugees at all border crossing points in Uganda. Many Ugandans also go to DRC to cultivate land and crops. In other words, there is a high level of cross-border movement and socializing. The main reasons for crossing the border are reported below (Table 3):

“They go [to Congo], but at the border there is no control.”

– Male participant, 37, Hoima District

**Table 3:** Movement across borders: Reported reasons for cross-border movement in Uganda.

*Reason for Crossing the DRC-Uganda Border*	*Sample Text*
Market days	*“*.* *.* *. *the fact that we live here*, *we always do shopping from Markets in Congo*.*”—*Kisoro District
Fishing	*“*.* *.* *. *these fishermen they are the ones who are at risk because they fish with the Congolese on the same waters and they share*, *they greet each other and all that*.*”*—Rukungiri district
*Boda boda* (motorcycle) driving	*“*.* *.* *.*we had a suspect in the hospital; somebody came from Congo… he came from Congo through Kyeshero* .* *.* *. *bleeding also but still taken by a boda boda”*—Kanungu District
Traders	*“Majority use boats to cross to Congo mainly to carry out business*, *for example buying chicken feeds*. *Though we also have a few who use vehicles*.*”*—Rukungiri District
Farming	*“We do farm in Congo*, *trade*, *and also Congolese cross to Uganda to do business and visits*.*”*—Kisoro District
Domestic water collection	*“Poor access to water is a very big problem here in Bunagana*. *Because of water shortage*, *we cross and fetch water from our friend’s homes in Congo*.*”*—Kisoro District

As reported above, participants crossed the border for primarily economic reasons, to engage in livelihood strategies such as fishing or market trading. Participants described crossing the border to seek more customers, because goods were generally expensive in the DRC. Bringing goods from Uganda to the DRC allowed traders to make a high profit. A concerning response was how commonly Bunagana (a border town) residents crossed into nearby DRC to fetch water. There were few sources of clean water in Bunagana, and crossing the border was a more practical option. In economically deprived areas with patchwork infrastructure, crossing the border is necessary for daily survival.

While the reasons are varied, officials (*n* = 64) and community members (*n* = 127) reported that border populations inter-marry, inter-trade, and inter-socialize. Indeed, as one participant stated: “we are one family, we are one tribe.” The porous nature of the border was observed on several occasions, where official points of entry (POE) were sparse. Meanwhile informal points of entry were more frequently used and included unmarked small roads or other land whereby individuals can cross into DRC from Uganda, and vice versa. Our research team conducted one FGD at an informal POE in Arua District, where men were seated near a small shop. Market traders prefer to cross via informal POE, where customs officials are not present and their goods will not be subject to additional charges (*Source*: FGD with men at a border district informal POE, Arua District).

### Livelihoods and cross-border movement

Livelihood needs drive cross-border movement and present a challenge to Ebola preparedness, prevention, and response. In this context, livelihood strategies refer to economic modes of subsistence including market trading, cash crop agriculture, fishing, transportation, or small businesses. Livelihood strategies among our participants ranged, from fishermen (*n* = 19) to farmers (*n* = 17), from long-distance truck drivers (*n* = 3) to *boda boda* (motorcycle taxi) drivers (*n* = 8). Livelihood seeking necessitates frequent cross-border movement, which Ugandans perceive as putting them at higher risk of contracting Ebola. Participants (*n* = 43) also described their economic precarity, and how low economic security necessitated extraordinary measures to engage in livelihood strategies. It is precisely because of this precarity that individuals are less able to make decisions about where and when to cross the border.

“Our border being porous, the Congolese are ever here and we are ever in Congo, they are ever in markets here and the Ugandans are ever in the markets of Congo. That means we are at higher risk.”

– Female participant, 24, Bundibugyo District

The study revealed that there was a high level of knowledge about Ebola—its origins, prevention, and treatment, and a high level of fear. Yet, changing behavior was weighed against the need to continue regular socioeconomic activities, much of which is subsistence work. In towns at the border of Uganda and the DRC, traders regularly cross the border to engage in market trading, and to obtain cheaper goods and items. Although long-distance truck drivers in Vurra, Northwest Uganda, are afraid of Ebola, they still have to cross deep into areas of the DRC in order to do their work. *Boda boda* (motorcycle taxi) drivers (*n* = 8) still carry dead bodies from the DRC, despite knowing the risks, because they have to earn a living and cannot refuse the extra money that such work pays. Indeed, participants described how *boda bodas* are the least costly way to transport a dead body. In Luweero, we interviewed one family that had multiple Ebola survivors (and deaths) because one son had transported a sick person in 2012 and who was later found to have Ebola.

“Those porous routes are there and they are very many. I was requesting our government to make a list of those pathways. We have a small road, and there is no one to make people wash hands.”

- Youth participant, 19, Kayonza Sub-County

Other participants reported how Ebola control measures hinder or restrict movement across borders and has infringed on individual decisions when to travel. Some may decide to proceed through informal POE to avoid Ebola surveillance activities at the formal POE. Therefore, livelihood strategies impact multiple aspects of Ebola prevention–from driving continued cross-border movement, to limiting cross-border movement (particularly through official POEs), to shifting decisions on how and when to cross the border.

### (Mis)trust in authorities and health workers

This final section details the ways in which rumors and information are spread across borders, exacerbating mistrust in Ebola preparedness and control efforts, and signifying general issues with trust in authorities. Additionally, we argue that it is political and economic factors that shape the levels of trust in health workers and other authorities. In districts with official POEs, trust in authority is hampered by a long history of fragile engagement between authority figures and community members in the borderlands. Participants (*n* = 13) reported perceived corruption such as paying bribes to border officials in order to transport goods, a practice which drove them to cross at informal POE. These economic transactions further degrade trust in officials, who are perceived to be working for their own benefit. The unintended consequence is that by crossing at informal POE, there is no Ebola screening of those individuals.

All FGDs (*n* = 25) and key informant interviews with local leaders (*n* = 9) and health workers (*n* = 12) raised issues of community (mis)trust in various authorities. Trust in local leaders was generally high, and local leaders were seen as a source of trusted information on Ebola prevention. Local leaders are also deeply engaged in their communities, involved in local issues and dispute resolutions, and they are historical sources of organization within Ugandan communities. On the other hand, trust in health workers was lower, with many community members complaining that government health workers in particular were not to be trusted. International and national NGO staff were regarded as an important source of support to communities, most likely because of the programs that they bring to communities. In fishing communities, there was generally a high mistrust in authorities, particularly the army. These nuances are discussed further below.

### Trust in local leaders

Local leaders, particularly Local Council I members (e.g. village chairpersons), are seen as trusted and integral components of a community's fabric. Where local leaders had been engaged, they were an important group to disseminate information on Ebola prevention and control—spreading messages about hand washing, screening, and how to report a suspected case. Meanwhile, in areas such as Arua and Pakwach, districts in North-Western Uganda, local leaders were described as not fully engaging with Ebola response activities.

“Village Chairperson talk about Ebola wherever they go, be it in church, be it in community or group gatherings for people to continue preventing Ebola.”

“Local leaders teach us to wash food items we buy from Congo before eating them. They also teach about Ebola during clan meetings.”

- Male participants, *Kisoro District*

### (Mis)trust in health workers

Perceptions about local government health workers had implications for trust and demonstrate low faith in the health system in general. As evidenced in the quote from Kagadi District, poor experiences with the health system are commonplace and contribute toward perceptions of neglect. Health facilities, meanwhile, are understaffed and undersupplied, especially in rural areas further from central Uganda. The consequence of this neglect is that rural residents do not choose a government health center as their first point of contact, often preferring private clinics (if they can afford them) or traditional healers. In one FGD in Pakwach District, 60% of individuals described having a poor opinion of government health workers. In another FGD in Kagadi District in Western Uganda, women described difficulties accessing health facilities which prevented them from seeking health care in time. Even if they do go to a government health center, it is often closed or under-staffed. These results demonstrate that the root causes of (mis)trust require a more substantial mode of community engagement than public education campaigns.

“What prevents us from seeking treatment for instance you go to Ndege (a government health facility) and you don’t find there the doctors, you take there the whole day suffering and you

end up coming back and the problem might worsen.”

- Female participant, 47, Kagadi District

### Trust in NGO workers

Participants mentioned the trusted work that NGO workers were doing to prevent Ebola in their areas. Often seen as impartial, apolitical mediators in the Ebola response, NGO workers were well-received by community members. Specifically, the work of the Red Cross, UNICEF, and the Infectious Disease Institute (IDI) were mentioned during interviews. These were all key players in the Ebola response in Uganda. However, some community members discussed how NGOs do not always "reach the ground." Community members wanted to be consulted on NGO priorities and asked for additional sensitization activities, additional information, and even Ebola vaccines. Ugandan policy to vaccinate high-risk health workers with the Ebola vaccine was widely publicized in 2018–19, and community members wanted to be included as well.

“There is Red Cross … they have put in much efforts, they are the ones I see who usually teach us, they are the ones doing most of the work … we want them so much because they help us a lot”

- Female participant, 32, Kanungu Disitrict

### Mistrust in authorities in fishing communities

Special attention must be paid to the dynamics in fishing communities, where trust in authority figures is very low, particularly trust in army soldiers. Due to the historical and political context in fishing communities, fisherfolk are wary of authorities after the military was described as seizing control of the fishing industry. Only regulated fishing boats were allowed on the waters, but these licenses were too expensive to obtain. Fishing communities were being asked to pay what they perceived as exorbitant fees to buy bigger boats, register existing boats, and pay for fishing licenses. Some participants (*n* = 6) described taking out loans to pay for these new licenses. Individuals reported getting deep into debt just to be able to conduct their daily work of fishing. It also meant that families could no longer fish for their household consumption, unless they had a licensed boat. In these areas, trust in authorities is very low and many participants even expressed their frustration in terms of one individual (quoted above) who wished that Ebola should come there and kill them, so that they do not have to pay back a loan they cannot afford. The participant above indexes deep mistrust and the reality that Ebola was not his most pressing concern, but rather it was the economic reality that shaped his day-to-day.

“… when I go to Congo, I don’t even think that Ebola is there because even if Ebola killed me, it would have saved me from that loan.”

- Male participant, 35, Rwenshama District

### Rumors across borders and (mis)trust in Ebola response

Participants in communities with high connectivity with Congolese counterparts described the spread of information and rumors across borders. In Kasese District, women (*n* = 8) described what they have heard from Congolese friends about the origins of Ebola–that it was started by the Congolese government to suppress opposition voters leading up to the December 2018 election in DRC. This demonstrates a diffusion of information across borders. Kasese District is also right across the border from where Ebola started in DRC. Participants with this point of view also reported being less likely to welcome or trust Ebola preparedness efforts. There was also a perception that Ebola was “over there” in DRC, and that on the Ugandan side, because there have not been many active cases, people are less at risk. One participant describes this:

“S*he said that the medicine of Ebola is waragi (local alcohol)*, *and on that side many people got Ebola but we who had stayed in bars the whole day drinking we never got Ebola she continued that even when doctors came they looked for us who were drinking and we were the ones to help the health workers*.*”*

- FGD, Female, 41, Rwenshama Landing Site

Communities that were closer to the source of the epidemic in DRC (e.g. Kasese District in Uganda) reported more rumors around Ebola; information was gained from their Congolese relatives or friends. Meanwhile, those who were further away from the epidemic reported fewer rumors.

## Discussion

Our research points to interrelated themes on Ebola in the borderlands of DRC-Uganda, from the perspective of 287 participants in moderate- and high-risk districts in Uganda. In the border context, communities are inter-linked and frequently travel across porous borders. Livelihood strategies and economic need necessitate cross-border travel, even when Ebola is present. The need to earn a living and survive is weighed against a perceived risk of Ebola, and these mental calculations shape individual decisions. Further, this constant cross-border movement has implication for the spread of information and lowered trust in Ebola response efforts. Our work demonstrates the importance of understanding the political economy of borderlands, where structural violence by the state contributes to community perceptions of neglect and disadvantage.

### Borders and livelihood strategies in a time of Ebola

This porous border facilitates constant cross-border travel, at both official and informal POE. Additionally, we have demonstrated how restricted economic security necessitates livelihood strategies, even during times of an epidemic. A basic lack of infrastructure or clean water means that community members have to cross the border to seek resources. The political-economic context shapes what economic opportunities are available, with a low number of jobs available in the formal economy. Individuals must instead rely on more informal work, such as market trading, and ultimately have less economic security in the long run. In contrast to previous work on livelihoods and Ebola, which looks specifically at the ways that Ebola hinders livelihood seeking [35], and the ways in which livelihood strategies make individuals more resilient [36], our work looks explicitly at livelihoods and cross-border travel with implication for Ebola control. In a context of Ebola preparedness, strategies should consider how individuals are constricted by economic fragility.

### Cross-border movement and (mis)trust in authorities in a time of Ebola

Our findings relate to previous anthropological work on community resistance to Ebola response, ultimately rejecting the term as other anthropologists have done [4,38,43]. Community resistance is a broader umbrella term that is polysemous and lacks sensitivity to political dynamics underlying this “resistance” [6]. By researching a marginalized context that has experienced structural violence [29], such as the fishing communities, this work conceptualizes state control over the fishing industry as a harm. It restricts fishing communities’ ability to provide for their households and to secure a stable source of food. In fishing communities, the combined lack of job opportunities and state control over fishing synergize to shape community mistrust of authorities. This research draws on structural violence theory to demonstrate the complex dynamics underlying trust.

Meanwhile, while work on trust has been conducted it also highlights the under-researched nature of this topic in an Ebola context [3,5]. Here, our work contributes to the literature on trust and Ebola by investigating the ways in which borderland dynamics in a marginalized setting shapes trust in authorities. In an area with a porous border, not only people move, but ideas and rumors cross borders as well. In a region where the health systems are under-resourced, trust in government health workers was lower compared to private health workers. The dynamics of trust are often driven by life in the borderlands, where trust is a complex issue shaped by a political-economic context of colonialism and postcolonial economic policies.

### Policy implications

Indeed, as has been noted by Chandler and colleagues [1], there are limits to correcting misinformation. Information must be placed into a political-economic framework, to better understand how it will be received by community members. Risk and prevention messages should be tailored to consider the dynamics of borderlands and should be delivered through appropriate channels. Specific recommendations geared toward Ebola policy and programming audiences are listed below:

- *Enhance sensitizing and training targeting individuals working in the transportation sector* (i.e. *boda boda* and truck drivers) to bring attention to the risk of transporting ill persons or dead bodies.
- *Train and engage more Village Health Workers (VHWs) to accompany Ebola response efforts at the community level*. Due to limited access to health care facilities, VHWs are critical to enhance Ebola preparedness in hard-to-reach areas. During an outbreak such as currently in DRC, neighbouring countries can consider increasing deployment of VHWs through tapping into volunteers trained specifically for Ebola or other such outbreaks.
- *Engage trusted groups in border districts*, including local leaders, NGO workers, private health workers, or religious leaders. These individuals should be engaged and supported to lead context-specific Ebola prevention activities.
- *Translate messages into local languages* and offer solutions, not just prescriptive "practices to avoid". In other words, due to significant barriers (e.g. livelihoods) to taking up Ebola prevention measures, it is important that we demonstrate ways to safely conduct burials, if possible. If we persist with a "stop" all behaviors approach, the uptake will be low and individuals may not take up recommendations.
- *Support economic well-being* approaches that bolster economic security, especially in the short-term during an epidemic. If a root cause of border crossing is livelihood, then the response should either support linkages to alternatives, or support mechanisms that secure (ideally both) informal and formal POEs.
- *Coordinate response efforts across borders*. This research demonstrates the interconnectedness of border regions. It is imperative that activities do not stop at the border, but specific coordination is in place to address the dynamic and porous border.

### Limitations and future directions

Our research focused on towns and border crossings. Additional research should focus on more rural areas in high-risk districts, beyond town centers. Many rural areas in the high-risk districts experience high traffic at informal border crossing points as community members go about their daily routines. Moreover, when persons crossing from DRC might avoid the formal border crossing points in towns, they resort to passing through rural areas with less monitoring. While it was important to engage local leaders in the process, we may have also oversampled from their networks. It would be important to include voices of those not connected to local leaders. Trust would also have implication for uptake of treatment, referral to Ebola Treatment Units, and uptake of vaccines. There are additional opportunities to build on research on those topics, similar to the work of other researchers [1–3]. Future research could be conducted over a longer period, using these findings as a foundation. Lastly, our work was conducted in a context of Ebola preparedness, before the outbreak crossed the border into Uganda. We were also time limited in conducting this research given the urgency due to the need to respond to an emergency. Trust is a difficult concept to operationalize. We encourage other researchers to build on our methods and findings to improve measurement of trust. We also see value in research that considers additional manifestations of trust, such as health-seeking behavior or movement across borders. Additional research on trust should be conducted on the DRC side, as well as in Uganda.

### Conclusions

In this paper, we investigated the borderland context of Ebola, with a particular focus on livelihood strategies, constraints, and (mis)trust in authorities. We have confirmed existing anthropological literature on border dynamics and how local context contours community responses to Ebola preparedness and control activities. Namely, communities with existing mistrust in authorities must be considered when designing more context-specific approaches. Additional work must be conducted to respectfully engage fishing communities, especially given the longstanding mistrust in government authorities. Ebola response activities should also consider the numerous informal points of entry along the porous DRC-Uganda border, where a majority of citizens cross routinely. The lack of surveillance activities in these areas portend negative consequences for current and future disease control efforts. Moreover, these findings should apply to other epidemic contexts, especially in borderlands. More research around policy and service delivery in border areas is urgent and critical to building effective public health strategies in border regions. Within this agenda, we argue that the efforts to strengthen linkages to communities during an Ebola response must be sensitive, locally responsive, and must promote meaningful partnerships.
